# *Menthol and Menthone* Associated with Acetylsalicylic Acid and Their Relation to the Hepatic Fibrosis in *Schistosoma mansoni* Infected Mice

**DOI:** 10.3389/fphar.2017.01000

**Published:** 2018-01-18

**Authors:** Karina A. Feitosa, Maurício G. Zaia, Vanderlei Rodrigues, Cynthia A. Castro, Ricardo de O. Correia, Fábio G. Pinto, Karina N. Z. P. Rossi, Lucimar R. S. Avó, Ana Afonso, Fernanda F. Anibal

**Affiliations:** ^1^Laboratory of Inflammation and Infectious Diseases, Department of Morphology and Pathology, Universidade Federal de São Carlos, São Carlos, Brazil; ^2^Faculdade de Medicina de Ribeirão Preto, Universidade de São Paulo, Ribeirão Preto, Brazil; ^3^Laboratory of Pathology, Department of Morphology and Pathology, Universidade Federal de São Carlos, São Carlos, Brazil; ^4^Departamento de Medicina, Universidade Federal de São Carlos, São Carlos, Brazil; ^5^Medical Parasitology Unit, Global Health and Tropical Medicine, GHTM, Instituto de Higiene e Medicina Tropical, Universidade Nova de Lisboa, Lisbon, Portugal; ^6^Bioanalytical, Microfabrication, and Separations Group, Instituto de Química de São Carlos, Universidade de São Paulo, São Carlos, Brazil

**Keywords:** *Schistosoma mansoni*, histology, inflammation, hepatic fibrosis, acetylsalicylic acid

## Abstract

Schistosomiasis is an important parasitic disease caused by *Schistosoma mansoni*, an intravascular trematode. Schistosomiasis treatment is limited to just one drug, Praziquantel (PZQ). Thus, studies on new antischistosomal compounds are of fundamental importance to disease control. Here we report on the effects of *Mentha piperita* L. compounds – menthol and menthone – in association with acetylsalicylic acid (ASA) in the regulation of hepatic fibrosis caused by schistosomiasis granulomas. Six different groups of Swiss rats were infected with 80 cercariae. Two groups received only menthol and menthol treatment at different concentrations (30 and 50 mg/kg); two groups received treatment with the same concentration of menthol and menthol, but associated the ASA. All groups received treatment for 14 consecutive days from the 35 days after the parasitic infection. In addition, three other groups were used: uninfected and untreated group, infected and untreated group and infected group treated with the commercial drug (single dose). Parasitological, cytological and histological analyses were performed. Results showed a significant reduction on the number of eosinophils found in the peritoneal cavity lavage (LPC) in all treated groups and on the number of eosinophils found in the blood of PZQ treated group, in the blood of the group treated with 30 mg/kg of Mentaliv^®^ and in the blood of group treated with 50 mg/kg Mentaliv^®^ + ASA when compared to the infected group. All treated groups presented a reduction in the parasite load, represented by the number of *S. mansoni* eggs, in the experimental group treated with 30 mg/kg of menthol and menthone a 62.80% reduction was observed and in the experimental group treated with 50 mg/kg of menthol and menthone + ASA a reduction of 64.21% was observed. In the liver histological analysis we observed that all Mentaliv^®^ treated groups expressed a unique cytological profile, with diffused cells through the granuloma. In the experimental group treated with 50 mg/kg of Mentaliv^®^ + ASA it was possible to observe the formation of type III collagen fibers, a typical wound healing characteristic. Our data strongly suggest that both the hepatic fibrosis and the inflammatory process were regulated through the schistosomiasis granulomatous process after treatment with menthol and menthone associated with ASA.

## Introduction

Schistosomiasis is a parasitic disease caused by various species of *Schistosoma* trematode worms and it is believed that more than 261 million people are affected worldwide (World Health Organization [WHO], 2015). Schistosomiasis occupies second position among tropical parasitic diseases, second only to malaria in Africa (Sarvel et al., 2011), affecting countries in Asia, South America and 90% of African continent (Menezes et al., 2012; Inobaya et al., 2014). In Brazil schistosomiasis is a parasitic disease caused by *Schistosoma mansoni*, an intravascular trematode (Gryseels et al., 2006). In Brazil, it is estimated that more than 25 million people live in endemic areas and more than 6 million people carry the disease (World Health Organization [WHO], 2008). Schistosomiasis at its chronic stage occurs when eggs are released by adult females in the mesenteric vessels, and they are carried to the liver sinusoids, where they get accumulated causing an inflammatory reaction (Pearce and Macdonald, 2002; Gryseels et al., 2006). Retained eggs, originate granulomas.

The granulomatous process corresponds to a secretion reaction of proteolytic enzymes by the *S. mansoni* eggs in blood, which leads to the induction and the mobilization of eosinophils, macrophages, lymphocytes, and plasmocytes. This process leads to the formation of collagen fibers that can promote the formation of fibrosis which then blocks pre-sinusoidal circulation, inducting to portal hypertension, collateral circulation, ascites, hepatosplenomegaly, and gastrointestinal varices (Van Der Werf et al., 2003; Gryseels et al., 2006).

Efforts to control schistosomiasis are based on an integrated effort of controlling the intermediate host, implementing sanitary education, increase sanitation coverage and patient treatment with Praziquantel (Chitsulo et al., 2000). For 40 years, Praziquantel (PZQ) has been used in trematodes and cestodes infections, being the only recommended drug against schistosomiasis. Both low activity against immature forms of the parasite and the appearance of *in vivo* and *in vitro* strains that are less sensitive to this drug (Novaes et al., 1999; Hagan et al., 2004; Frezza et al., 2007; Pinto-Almeida et al., 2015), highlights need for PZQ alternatives. *Mentha piperita* L. is popularly known as peppermint has been extensively studied due to its therapeutics potential. *M. piperita* L. has been described in the literature to cause several alterations on the surface plasma membrane of *Giardia lamblia* trophozoites, revealing the antiprotozoal potential of *M. piperita* L. (Vidal et al., 2007). Furthermore, essential oils of *M. aquatica, M. longifolia* L., and *M. piperita* L. have shown bactericidal activity against *Escherichia coli* and also antifungal activity (Neda et al., 2003).

Acetylsalicylic acid (ASA) is largely used as an anti-inflammatory drug. ASA was synthesized by using salicin extracted from *Salix alba*. ASA is classified as non-steroidal anti-inflammatory drug and is used as antipyretic, anti-inflammatory, analgesic and antiplatelet aggregation agent (Patrono et al., 2005). It is known that parasites such as *Ascaris* and *S. mansoni* make use of prostaglandins synthesis to, establish infection in the host and that ASA inhibits the action of cyclooxygenase (COX), causing it to cease production of prostaglandins (Noverr et al., 2003; Patrono et al., 2005; Hilário et al., 2006; Kubata et al., 2007).

A previous study of our group using mice infected with *S. mansoni* and treated with *M. piperita* L. ethanolic extract, revealed a decrease in egg counts in the intestine, liver, and feces of the animals (Dejani et al., 2014). Another study of our group showed that the doses used here for menthol and menthone (30 e 50 mg/kg) are the most promising for the search for a new compound that could be used as a phytotherapeutic agent in the control of schistosomiasis (Zaia et al., 2016).

In this context, we evaluated the effect of a combination of menthol and mentone compounds obtained from *M. piperita* L. (a commercial drug called Mentaliv^®^) at doses of 30 and 50 mg/kg with ASA, seeking to improve the anti-inflammatory effect, promoting regulation in the formation of granulomas leading to the formation of hepatic fibrosis, using an experimental murine model for schistosomiasis. The methods and some results obtained can be seen in the graphic abstract (Image 1) of the Supplementary Material.

## Materials and Methods

### Animals

Female swiss mice with 6 weeks and weighing between 16 and 20 g were obtained from the animal facilities at the Universidade Federal de São Carlos (UFSCar). These animals were maintained in the Laboratory of Inflammation and Infectious Diseases of the Department of Morphology and Pathology of UFSCar in experimental conditions with free access to food and water. The Ethics Committee on animal use from this University, CEUA UFSCar n° 009/2014, approved the project.

Mice were randomize using physical randomisation, once the animals necessary to the different treatments (each experiment: six animals per treatment, except negative control, with five animals), were obtained a number was written on 82 pieces of paper, these are folded and placed in a receptacle which is shaken. A paper is withdrawn, and the first animal is assigned to the indicated treatment, and so on. This was done at a desk by recording animal number and assigned treatment on paper before going to the animal house. Once randomization was done, their allocation concealed, and blind outcome assessment was used. Animals were obtained in two different lots. Each independent batch was used to perform each one of the independent experiments (duplicate: n total: 41 × 2 = 82 animals). The animals were separated into different experimental groups as shown in **Table 1**.

**Table 1:** Experimental groups using *Swiss* mice in two independent experiments.

Group	Mice (*n*)	Dose	Treatment (35 days
			after infection)
(1) Negative control	05	–	–
(2) Positive control^∗^	06	–	–
(3) Praziquantel^∗^	06	400 mg/kg/0,2 mL	Single dose
(4) Menthol and menthone 30**^∗^**	06	30 mg/kg/0,2 mL	14 days treatment
(5) Menthol and menthone 50**^∗^**	06	50 mg/kg/0,2 mL	14 days treatment
(6) Menthol and menthone 30 + ASA**^∗^**	06	30 mg/kg of menthol and menthone + 100 mg/kg of ASA/0,2 mL	14 days treatment
(7) Menthol and menthone 50 + ASA**^∗^**	06	50 mg/kg of menthol and menthone + 100 mg/kg of ASA/0,2 mL	14 days treatment


*^∗^Mice infected with *S. mansoni* cercariae.*

### Infection

Mice were infected with 80 cercariae of *S. mansoni*, lineage LE, kept at the Department of Biochemistry and Immunology, Ribeirão Preto Medical School, University of São Paulo (USP). The cercariae were previously counted in optical microscope. Mice were infected subcutaneously with cercariae of *S. mansoni* were inoculated about 80 cercariae/0.3 ml saline (NaCl 0.9%)/animal, using a final volume of 1 mL syringe (Dejani et al., 2014).

The *S. mansoni* requires BSL-2 and ABSL-2 practices, containment equipment and facilities are recommended for laboratory work with infective stages of the parasites. Gloves were used when there was direct contact with water containing cercariae. Long-sleeved laboratory coats or other protective garbs were worn when working in the immediate area.

### Treatment

For the herbal treatment we used a commercial product Mentaliv^®^ (Aspen Pharmaceutical; Lot 11M20A01), comprising menthol (30–55%) and menthone (14–32%) presented in a gastro resistant capsule containing 200 mg of active principal. Mentaliv is prepared from *M. piperita* L. For the treatment the capsule was solubilized in filtered water and therefore administrated by gavage at a dose of 30 mg/kg or 50 mg/kg. The ASA was obtained from macerated child Aspinin^®^ tablets, (100 mg) (EMS Laboratory). Daily ASA was diluted in 1 mL of filtered water and associated with menthol and menthone treatment.

Praziquantel was obtained from macerated tablets of Tenil^®^ Vet tablets (50 mg; Lot 3489101) solubilized in Dimethyl Sulfoxide 3% (DMSO), administered in a single dose of 400 mg/kg (Dejani et al., 2014). Positive control group was infected and received no treatment, while the negative control group was neither infected and nor treated. After 35 days of infection with *S. mansoni* cercariae, the herbal treatment was carried out for 14 consecutive days, being given a final volume of 0.2 mL per treatment. Water and food were removed 1 h before treatment administration. Experiment was performed in duplicate, with experimental groups of 5–7 mice. On day 49 post parasite infection mice were euthanized using a CO_2_ chamber.

### Parasitological Parameters

The eggs were counted using the protocol adapted from Katz and Peixoto (2000), which is determined by the number of eggs/gram of feces from rats. The Test Helm kit (Bio-Manguinhos FIOCRUZ) was used. Fresh feces were used and the test was performed using one pool per group. On the 48th day post parasite infection and for 24 h each animal was individually housed a cage. In this way, we obtained stool sample from each animal. Afterward, a homogenization of all the feces belonging to each pool was performed, the slide was then prepared. The feces were screened in nylon and the amount of feces determined by filling an orifice with a known diameter placed on a glass slide were thus coated with green cellophane coverslip impregnated with a green solution of malachite 3%) for preservation and bleaching of the material. The slides were read and the eggs were counted using an optical microscope.

The number of eggs was calculated using a standard formula for the Helm Test Kit, Bio-Manguinhos: number of eggs counted × 24 = eggs/gram of feces. The reduction in egg numbers was determined by comparing the mean number of eggs recovered from each experimental group and the mean number of eggs from the positive control group, according to the formula:

$$DR=\frac{(RCG-REG)\times 100}{RCG}$$

where DR is the degree of reduction, RCG is the number of eggs in the positive control group and REG, number of eggs in the experimental group (Fonseca et al., 2004).

### Leukocyte Profile: Eosinophils

#### Peritoneal Cavity Lavage (LPC) and Blood

Blood eosinophils were washed from the peritoneal cavity (LPC) of the animals in all experimental groups 49 days post parasite infection and analyzed. Total number of eosinophils/mm^2^ in both compartments (blood and LPC) was determined using Turk’s solution to lyse RBC at a 1:20 dilution. Each sample was counted using a Neubauer chamber. Blood smears were used to count differential blood cell. LPC and blood slides were stained using Panotic-Laborclin dye and 100 cells were counted, being differentiated in eosinophils, using light microscopy, with a final amplification of 1000× (Nikon alphaphot 2 - ys2).

### Histology

Mice liver was removed on day 49-post parasite infection and fixed in 10% formalin. Specimens were embedded into paraffin blocks, sectioned into sections of 5 μm and stained with Sirius Red and Haematoxylin-Eosin for histological analysis under light microscopy. The slides were prepared in the Pathology Laboratory, Department of Morphology and Pathology (DMP) UFSCar. Slide reading and photographs were held in the Department of Medicine (DMED) UFSCar. A microscope (Olympus U-MDOB3) containing coupled camera (Olympus SC30), and polarized lens required for the slides stained with Sirius Red was used.

### Statistical Analysis

Leukocyte profile results were expressed as mean ± SD. Results were analyzed through the parametric test One-way ANOVA (*One-Way analysis of variance*), *post hoc* analysis by Tukey Method (*Compare all pairs of variables*). Statistical significance was set at *p* < 0.05.

## Results

### Parasitological, Cytological, and Histological Analysis

**Table 2** shows the results from Kato-Katz assay and the degree of reduction in egg numbers.

**Table 2:** Mean number of eggs/gram of faeces and degree of reduction on egg numbers, after treatment (49 days post parasite infection).

Experimental group	Mean number off eggs per group (eggs/gram of feces)	Degree of reduction (DR) in egg numbers in %
Positive control	1140	–
Praziquantel	52	95.44%
Menthol and menthone 30	424	62.80%
Menthol and menthone 50	680	40.35%
Menthol and menthone 30 + ASA	672	41.05%
Menthol and menthone 50 + ASA	408	64.21%


**Table 3** shows the results obtained by histopathological analysis of the mice liver using both stains (HE and Sirius red staining). Other data referring to **Table 2** can be seen in Supplementary 1, 2.

**Table 3:** Histopathological analysis.

Group	Haematoxylin-eosin	Sirius red
Negative control	Regular parenchyma	Collagen deposition only around the vessels

Positive control	Hepatic granulomas and mixed lymphocyte infiltration	Granulomas surrounded by a big amount of collagen

Praziquantel	Hepatic granulomas and infiltration	Smaller granulomas, with more clear fibers (greenish)

Menthol and menthone 30	Hepatic granulomas and infiltration, but it was possible to observe that all menthol and menthone (Mentaliv^®^) treated groups expressed a unique cytological profile, whose cells were diffused through the whole granuloma	Collagen with thicker and darker fibers was observed, only in the granulomatous areas
Menthol and menthone 50		
Menthol and menthone 30 + ASA		
Menthol and menthone 50 + ASA		


**FIGURE 1:**
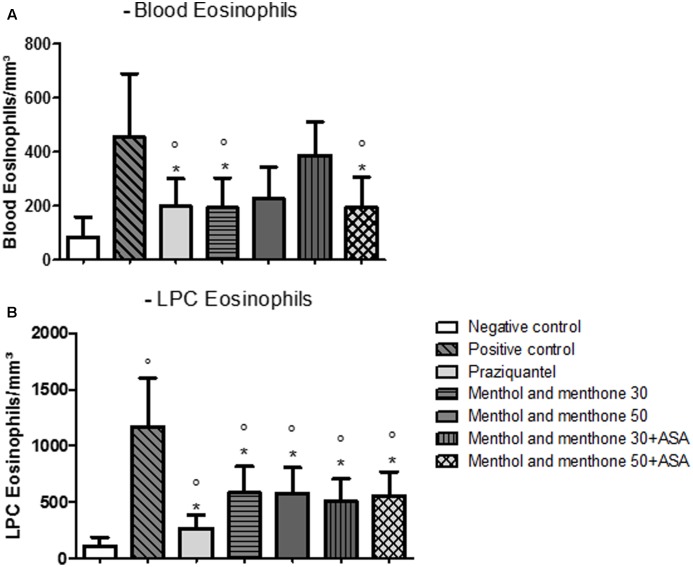
Number of eosinophils in the blood and LPC 49 days post infection with *Schistosoma mansoni*. **(A)** Blood eosinophils **(B)** LPC eosinophils. (°) There is a statistically significant difference between the results of this experimental group when compared to the control group. (^∗^) There is a statistically significant difference between the results of this experimental group when compared to the infected untreated group. Data is presented as mean ± SEM. The difference was considered significant when *p* < 0.05.

**Figure 1** shows the reduction in the number of eosinophils in the blood and LPC of infected animals either treated or not treated. The eosinophils found in the blood of PZQ treated group, 30 mg/kg menthol and menthone (Mentaliv^®^) treated group and 50 mg/kg menthol and menthone (Mentaliv^®^) + ASA treated group presented a significant reduction in the total number of these cells when compared to the infected group (**Figure 1A**). In the PZQ treated group there was a reduction of 56.2%, also in the experimental group with the combination treatment of 50 mg/kg menthol and menthone (Mentaliv^®^) + ASA there was a reduction of 57.4% and for the group treated with 30 mg/kg menthol and menthone (Mentaliv^®^) there was a 57.6% reduction when compared to the Positive control group. With respect to LPC eosinophils, all treated groups presented a significantly reduction in eosinophils numbers when compared with the infected untreated group (**Figure 1B**).

**FIGURE 2:**
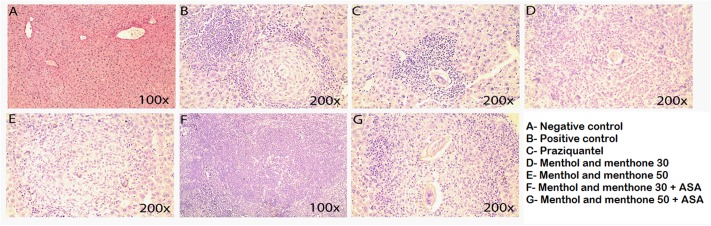
Liver histology stained with HE. Negative control **(A)**, positive control **(B)**, praziquantel **(C)**, menthol and menthone 30 **(D)**, menthol and menthone 50 **(E)**, menthol and menthone 30 + ASA **(F)** and menthol and menthone 50 + ASA **(G).** Coloration hematoxylin-eosin (H.E) increase: 100×/color H.E. Increase: 200×; except **(A,F)** with 100× magnification.

**FIGURE 3:**
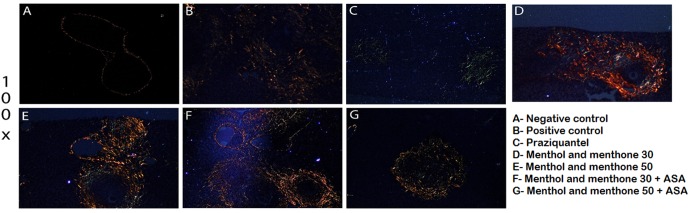
Liver histology stained with Picro Sirius Red. Negative control **(A)**, positive control **(B)**, praziquantel **(C)**, menthol and menthone 30 **(D)**, menthol and menthone 50 **(E)**, menthol and menthone 30 + ASA **(F)** and menthol and menthone 50 + ASA **(G)**. Picro-Sirius Red/Increase: 100×.

Livers of infected + treated or untreated animals were analyzed 49 days post parasite infection using: HE (for inflammation detection) and Sirius red (for collagen identification). In addition to histopathological analysis, data from parasitic load and total number of eosinophils after treatment were used to understand menthol and menthone (Mentaliv^®^) action as well as its association with ASA during *S. mansoni* infection using mice experimental model. Using both colorations, mice livers from animal of the negative control group presented its preserved histology. Using HE stain, the parenchyma was normal and there was no granulomas, using Sirius red staining collagen deposition around vessels was observed as expected (**Figures 2A**, **3A** and **Table 3**). When using HE staining in the positive control group (infected with S. mansoni and untreated) we observed the presence of liver granulomas and mixed lymphocyte infiltrate (macrophages, eosinophils, neutrophils, lymphocytes), while using Sirius red staining we observed granulomas with large amounts of collagen around. The positive control group was the group with the highest number of eggs/gram of feces (**Figures 2B**, **3B** and **Tables 2**, **3**). Praziquantel experimental group had the highest degree of reduction in egg numbers – 95.44% (**Table 2**) and using Sirius red coloration we were able to observe lighter fibers (greenish) apparently thinner and possibly a type III collagen (**Figure 3C** and **Table 3**). All groups treated with menthol and menthone (Mentaliv^®^) presented the same profile of cellular infiltrate, similar to the group treated with PZQ when using HE staining (**Figure 2C**), but its constituents, granular leukocytes were found more widespread throughout the granuloma (**Figures 2D–G** and **Table 3**) in the groups treated Mentaliv^®^. Using Sirius Red staining in the group treated with 50 mg/kg menthol and menthone (Mentaliv^®^) + ASA (**Figure 3G** and **Table 3**) although it was possible to observe the presence of larger granulomas when compared to Praziquantel group it was also possible to observe a necrotic center with lighter fibers (greenish) when compared to the otherwise darker fibers (orange), observed in the Praziquantel experimental group, suggesting a possible increased in the presence of type III collagen, which is present in the healing processes. In addition, among the Mentaliv^®^ treated groups we were able to observe some degree of egg count reduction as seen in **Table 2**. In the menthol and menthone – Mentaliv^®^ treated groups (**Figures 3D–F**) we were able to detect the presence of a collagen deposit with thicker and darker fibers only in the granulomatous areas using Sirius Red staining.

## Discussion

Polyphenolic compounds present anthelmintic activity and there is evidence that menthol and menthone have antimicrobial effects (Iscan et al., 2002; Deore et al., 2009). Niclosamide, a synthetic phenolic compound, can act in helminths, interfering in the oxidative phosphorylation, thus affecting energy generation and interfering in the parasite’s metabolism (John et al., 2009). Flavonoids, mainly eriocitrin, narirutin, hesperidin, and luteolin 7-*O*-rutinoside are also found in *M. piperita* L., and present great pharmacological potential (Fecka et al., 2004). All these substances, which are present in the *M. piperita* L. ethanolic extract, can be responsible for this plant’s antiparasitic potential. Thus, the Mentaliv^®^ capsule used in this study might not have had completely effective action, since it contained only menthol and menthone in its composition.

The ASA, besides its anti-inflammatory, antipyretic and anti-rheumatic effects, is an immune-modulator, although it is not known by this function. Previous studies described its property on lymphocyte modulation and it is believed that its action is due to the leukotrienes metabolism inhibition (Sainte-Laudy, 1997). Another aspect is that the ASA’s main anti-inflammatory action is due to its cyclooxygenase (COX) inhibitory effect, ceasing the prostaglandins productions, which are responsible for the pain and inflammation processes (Patrono et al., 2005). Helminthic infections are unavoidably associated to high prostaglandins production, which engage with the host immunological response, leading to the infection symptoms such as sensibility to pain and fever (Noverr et al., 2003; Hilário et al., 2006). Studies by Kubata et al. (2007), describe prostaglandins production in *S. mansoni, Ascaris* spp., and *Onchocerca volvulus* infections. Hosts are not the only ones performing prostaglandins synthesis, but the parasites themselves have a mechanism that allows these molecules’ biosynthesis (Kubata et al., 2002, 2007; Sánchez-Ramírez et al., 2004). In this context, it is suggested that ASA is related to the decrease in prostaglandins production and, consequently, in the inflammation inhibition, because prostaglandins can be one way by which parasites modulate its hosts’ immune response, thus establishing the characteristic infection pattern of the disease.

Eosinophils actively participate in the immune response of parasitic diseases, mainly the ones caused by helminths. These leukocytes secrete proteins, which damage the parasites; the major basic protein (MBP), the eosinophil cationic protein (ECP), the eosinophil peroxidase (EPO), and the eosinophil-derived neurotoxin (EDN) are examples of these proteins (Swartz et al., 2006). Granulomas, present in schistosomiasis infection are 50% composed of eosinophils (Swartz et al., 2006). Lins et al. (2008), in an *in vivo* study, demonstrated that these cells were found differently expressed and distributed in each hepatic granuloma stage.

Leukocytes and fibrotic population cells profile change gradually according to the granuloma evolution. In the pre-granulomatous stage, a small number of eosinophils are found around the eggs, in the exudative pre-granulomatous stage, there is an increase in the number of eosinophils in the granuloma. In the necrotic-exudative stage, eosinophils concentrate around and in the center of the granuloma, being scarce in necrosis areas. In the productive stage, collagen fiber formation begins and eosinophils are still found all over the granuloma. Lastly, in the fibrosis healing stage, eosinophils are found around and in the center of the granuloma, like in the necrotic-exudative and the productive stages, however, in larger numbers. Concerning the results on eosinophilia numbers, drug treatments significantly lead to its reduction. Concerning blood eosinophils, the experimental groups treated with PZQ, 30 mg/kg of menthol and menthone (Mentaliv^®^) and the 50 mg/kg menthol and menthone (Mentaliv^®^) + ASA groups presented a significant reduction in these cells total numbers when compared to the Positive Control group (**Figure 1A**). When analyzing LPC, all treated groups presented a significant reduction on eosinophils when compared to the positive control group (**Figure 1B**). In a previous study from our group in an *in vivo* assay (*M. piperita* L. extract) we observed an increase in the number of eosinophils in *S. mansoni* infected and untreated mice, a result very similar to the one obtained in this work, where the infected but non-treated group presented the highest number of eosinophil (Dejani et al., 2014). Treated animals (both with PZQ, menthol and menthone and menthol and menthone + ASA) presented a reduction in eosinophils both in LPC and blood. Thus, our results contribute to the understanding that eosinophils may be important factors for the development of pathophysiology in schistosomiasis, and their reduction by the proposed treatments have a good prognosis for the control of this disease. Therefore these results we can suggest that will modulate the development of hepatic fibrosis in these animals, whereas subsequent histological analyses show in a more significant deposition of collagen repair.

The histopathological analyses in this work found some differences in the treated and the untreated infected animal’s livers. Using H.E stain, it was possible to observe granulomas, lymphocytic infiltrates, lymphocytic halos, inflammation and lymphocytic components. Previous data from our research group showed that *M. piperita* plant extract promotes integrin modulation contributing to recruitment, particularly of eosinophils and lymphocytes T CD_4_^+^ (Dejani et al., 2014). The proposal to use a drug containing the main components of the plant associated with the AAS is of great importance to the search for more effective drug for both infection control and for its anti-inflammatory activity.

The production of prostaglandins in the inflammatory process is a very important factor for maintaining the production of cytokines and other mediators related to tissue inflammation, as well as regulation of molecules involved in the recruitment of cells that will form granuloma. COX-2 is the primary enzyme involved in the metabolism of arachidonic acid (Ricciotti and Fitzgerald, 2011). Thus, understanding tissue changes, allows us to see the effect of an anti-inflammatory already known, associated with this drug for the control of inflammation, especially the liver during murine schistosomiasis.

Therefore, histopathological analysis with evaluation of inflammation and collagen deposition becomes important in this study. Sirius Red staining method allows collagen type differentiation coloration. This is due to the Sirius Red F3AB dye reaction, which increases the collagen fibers’ birefringence when in contact. Type I collagen shows yellow, orange or red colors and thicker shape. Type III Collagen shows greenish color and thinner fibers (Junqueira et al., 1982). Collagen fiber deposition analyses was done only qualitatively, observing collagen fibers’ injury, color and thickness, making necessary quantitative analyses demonstrating the type of involved collagens in each observed slide and the collagen deposition percentage, providing analysis to verify fibrosis focuses on the development of experimental schistosomiasis.

In the Praziquantel treated group (**Figure 3C**) it was possible to observe the presence of smaller granulomas, clearer fibers (greenish), apparently thinner and possibly a type III collagen. In the experimental group treated with 50 mg/kg of menthol and menthone (Mentaliv^®^) and in the experimental group treated with the combined treatment with menthol and menthone (Mentaliv^®^) and ASA (**Figure 3E**), we observed a necrotic center with more clearer fibers (greenish) than darker fibers (orange), suggesting the presence of a type III collagen. Type I collagen is the most abundant. It’s found in the deepest skin layer, the reticular dermis, and its total diameter is 1–20 μm (Kuhn, 1988). Type III is more superficially located, in the papillary dermis, and its diameter is 5–2 μm. In cicatrisation processes, type III collagen is in higher proportion (Kuhn, 1988; Broughton et al., 2006; Isaac et al., 2010). In the experimental group treated with 50 mg/kg of menthol and menthone (Mentaliv^®^) + ASA we can observe a larger presence of type III collagen fibers, typically observed on healing processes and also on cell broadcast granulomatous in all groups treated with menthol and menthone (**Figure 3G**).

In the study by Zaia et al. (2016) from our research group, trials for treatment with menthol and menthone were performed in two different ways. Treatment after confirmation of infection was done 15 days post drug treatment and 60 days treatment was performed from the 1st day of infection. However, prolonged treatment was better, but we know that shorter treatment times are often more successful for the lower drop-out of therapies. Therefore, in this model we reduced the dose and we kept the dose that was effective. However, only the treatment with menthol and menthone used by Zaia et al. (2016) was not 100% effective for the control of schistosomiasis. Therefore, we prefer to associate ASA and to verify synergism in this treatment. Other aspect to be considered is that in the Zaia et al. (2016) model Balb/C animals that are isogenic were used. And in this study mimicking the population we use the swiss, which is heterogenic and can show more clearly the effect of treatments on a population.

Our results suggest that components menthol and menthone of *M. piperita* L. acts in the modulation of the inflammatory process during disease (granulomas) and in combination with ASA might help during the hepatic inflammation and fibrose control in the model of murine schistosomiasis.

## Author Contributions

KF: main author of the work. She participated in all the techniques and analyzes of this work. She made graphs, tables, figures, and participated in the writing of the article. MZ: he actively participated in the techniques developed. VR: realization the infection of mice with *S. mansoni* in his laboratory. He participated in the analysis and discussion of the results this step. CC: participation active in histological techniques. She participated in the discussion of the results this step. RC: participation in the editing of the histological images and participated in the writing of the article. FP: availability of the necessary equipment for technique of preparation of histological slides and colorations. He participated in the analysis and discussion of the results this step. KR: availability of the necessary equipment for technique of preparation of histological slides and colorations. She participated in the analysis and discussion of the results this step histological slide. LA: participation in the reading of histological slides. She participated in the discussion of the article in that part. AA: she participated in the analysis, discussion and formulation of the results and in the writing of the article. FA: main advisor of project. She actively participated in the work and in the techniques developed. She participated of the analysis, discussion and formulation of the results and in the writing of the article.

## Conflict of Interest Statement

The authors declare that the research was conducted in the absence of any commercial or financial relationships that could be construed as a potential conflict of interest.
