# Supplementary material for: Menthol and Menthone Associated with Acetylsalicylic Acid and Their Relation to the Hepatic Fibrosis in Schistosoma mansoni Infected Mice
Source: Front Pharmacol. 2018 Jan 18;8:1000. doi: 10.3389/fphar.2017.01000 (PMC5778335; doi:10.3389/fphar.2017.01000)
Supplement: Supplementary file 1 [file Data_Sheet_1.docx]

**Supplementary**

**Image supplementary 1:** Graphics abstract of article

**
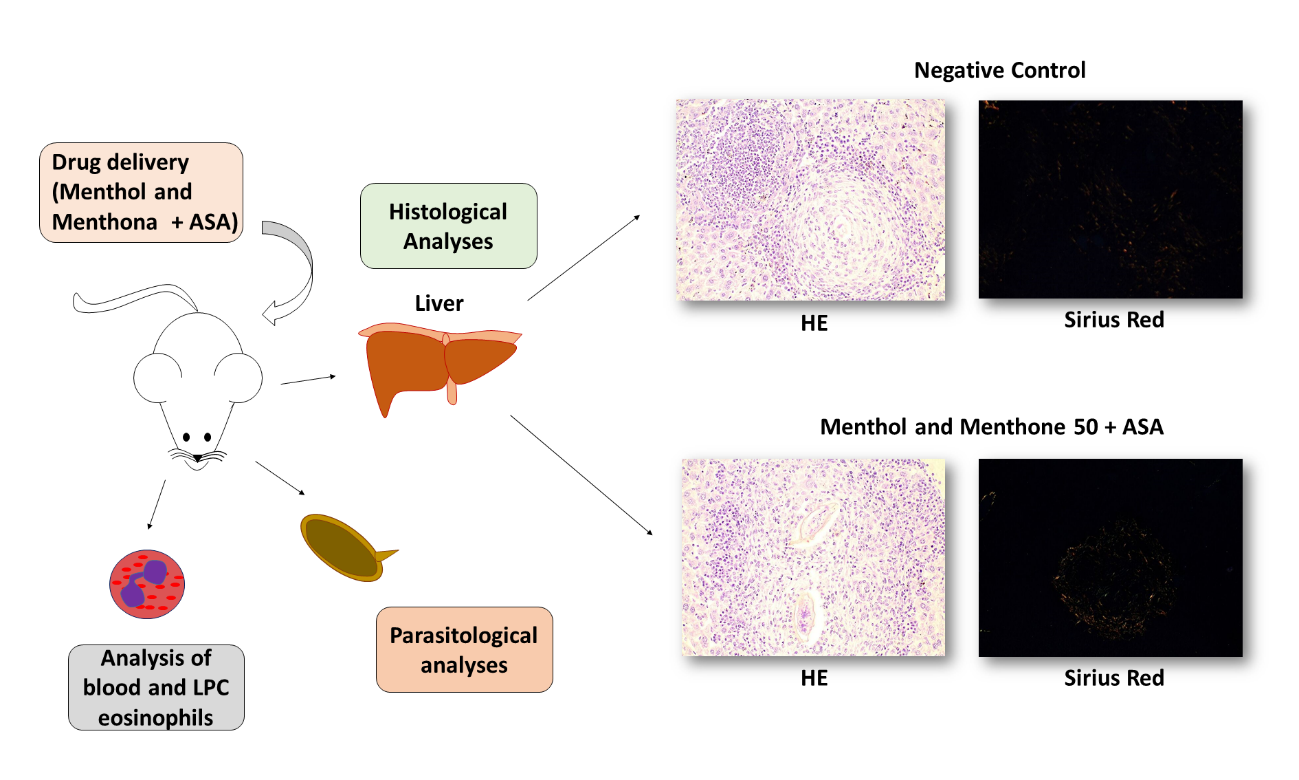
**

**Image supplementary 1:** Methodologies and main results obtained in the article: “Menthol and menthone associated with acetylsalicylic acid and their relation to the hepatic fibrosis in *Schistosoma mansoni* infected mice”.

**Supplementary 1:** Number of eggs / gram of faeces

**Supplementary 1:** Number of eggs / gram of faeces 49 days post treatment. Data represents the number of eggs counted on each slide for each group (pool) multiplied by a factor of 24.

| **Supplementary 2: Additional Kato-Katz information** | | | |
| --- | --- | --- | --- |
| **Experimental**  **Group** | **Number of eggs counted per slide (pool / group)** | **Number of eggs x 24 (eggs/gram of faeces)** | **Mean number off eggs per group**  **(eggs** **/gram of faeces)**  **n=6\group** |
| Positive control | 285 | 6840 | 1140 |
| Praziquantel | 13 | 312 | 52 |
| Menthol and menthone 30 | 106 | 2544 | 424 |
| Menthol and menthone 50 | 170 | 4080 | 680 |
| Menthol and menthone 30 + ASA | 168 | 4032 | 672 |
| Menthol and menthone 50 + ASA | 102 | 2448 | 408 |

**Supplementary 2:** Absolute numbers obtained during Kato-Katz test.
